# Why are all phase resetting curves bimodal?

**DOI:** 10.1186/1471-2202-14-S1-P398

**Published:** 2013-07-08

**Authors:** Sorinel A Oprisan, Davy Vanderweyen, Patrick Lynn, Derek Russell Tuck

**Affiliations:** 1 Department of Physics and Astronomy, College of Charleston, Charleston, SC 29424, USA; 2 Department of Computer Science, College of Charleston, Charleston, SC 29424, USA

## 

Neurons are excitable cells, which means that they operate close to a bifurcation points that allows them to switch back and forth between stable steady states and periodic attractors [1]. Repetitive firing neurons belong to either Class 1, i.e., characterized by a continuous frequency versus bias current (f-I) curve and arbitrarily low frequency of oscillation, or Class 2, i.e., discontinuous f-I curve and firing starts with a relatively high frequency at threshold stimulation [2]. The bifurcation mechanism leading to repetitive firing is related to excitability classes, i.e., saddle node on invariant circle (SNIC) bifurcation is associated to Class 1 and Andronov-Hopf (HB) bifurcation to Class 2 excitability [3]. The complexity of model neurons can be significantly reduced near bifurcation points, e.g., by using normal forms. Although such dramatic reductions oversimplify biophysical mechanisms they allow a better understanding of the phenomenology of phase locked modes and synchrony in neural networks. A phase resetting curve (PRC) is not concerned with the biophysical details of neurons, but rather tabulates the transient changes in the firing period due to an external perturbation, usually a brief current or synaptic conductance pulse. Despite its limitations, PRC-based approaches to treating Parkinson's and epilepsy are at the forefront of practical applications [4], or predicting the shape and timing of necessary stimuli that destabilize a synchronous state of a large population of neurons [5].

It was long advocated that the PRCs are either of Type I when the bifurcation mechanism leading to oscillatory behavior is a SNIC, or of Type II when the bifurcation is of HB type [3,6]. Type II PRC has an almost sinusoidal shape and comparable sizes of the positive and negative lobes, and synchronize more readily due to their ability to both slow down and speed up [3,6]. Type I PRC has a disproportionate ratio of the two PRC lobes, can predominantly speed up (or only slow down) their rhythm when coupled with other neurons.

We showed that any stable limit cycle oscillator has two remarkable points, which we called neutral points, at which brief perturbations do not induce any phase resetting. Furthermore, for a planar system there are only two such neutral points and we derived an analytical method of localizing them based on the Floquet multipliers. The PRC changes its sign at neutral points. Since there are two neutral points for any planar system it results that the corresponding PRCs must be all bimodal. Additionally, we derived analytical criteria for the degree of bimodality and were able to smoothly control the transition of the PRC from a Type I to a Type II. The ability to smoothly change the PRC type through experimentally available control parameters, such as bias currents or pharmacologically controlled ionic channels, has potential application in shaping the activity of neural cells and networks.

## References

[B1] Izhikevich EM Dynamical Systems in Neuroscience: The Geometry of Excitability and Bursting 2007 Cambridge, Mass: MIT Press

[B2] Hodgkin AL The local electric changes associated with repetitive action in a non-medullated axon J Physiology 1948 107 165 181 16991796 10.1113/jphysiol.1948.sp004260 PMC1392160

[B3] Ermentrout GB Glass L Oldeman BE The shape of phase-resetting curves in oscillators with a saddle node on an invariant circle bifurcation Neural Comput 2012 24 3111 3125 10.1162/NECO_a_00370 22970869

[B4] Abbott LF van Vreeswijk C Asynchronous states in networks of pulse-coupled oscillators Phys Rev E 1993 48 1483 1490 9960738 10.1103/physreve.48.1483

[B5] Schnitzler A Gross J Normal and pathological oscillatory communication in the brain Nat Rev Neurosci 2005 6 285 296 15803160 10.1038/nrn1650

[B6] Tass PA Desynchronization of brain rhythms with soft phase-resetting techniques Biological Cybernetics 2002 87 2 102 115 10.1007/s00422-002-0322-5 12181586

[B7] Gutkin BS Ermentrout GB Reyes AD Phase-response curves give the responses of neurons to transient inputs Journal of Neurophysiology 2005 94 2 1623 1635 10.1152/jn.00359.2004 15829595

